# Material Extrusion of the Poly(lactic acid)/HAp Nanocomposite Scaffold for Bone Tissue Applications: A Comprehensive Investigation

**DOI:** 10.1021/acsomega.5c02055

**Published:** 2025-09-16

**Authors:** Arunkumar Thirugnanasambandam, Ramasamy Nallamuthu, Sanjay Mavinkere Rangappa, Suchart Siengchin, Vishnu Vijay Kumar

**Affiliations:** 1 Centre for Sustainable Material and Surface Metamorphosis, 560142 Chennai Institute of Technology, Chennai 600069, India; 2 Natural Composites Research Group Lab, Department of Materials and Production Engineering, The Sirindhorn International Thai-German School of Engineering (TGGS), 67994 King Mongkut’s University of Technology North Bangkok (KMUTNB), Bangkok 10800, Thailand; 3 Structural Engineering, Division of Engineering, 167632 New York University Abu Dhabi (NYUAD), Abu Dhabi 129188, United Arab Emirate

## Abstract

The material extrusion of biodegradable composite scaffolds has emerged as an alternative for addressing the existing challenges associated with bone regeneration. The present study aims to fabricate a 3D-printed bioscaffold to facilitate bone regeneration by interacting with the local extracellular matrix and providing load-bearing capability. Hence, a biodegradable PLA blended with hydroxyapatite (HAp) bioceramic material has been utilized for providing essential properties for bone regeneration applications. The various PLA/HAp blend filaments were fabricated at multiple blending ratios (94/6, 88/12, and 82/18) to examine the most appropriate blend ratio for providing the intended applications. EDAX analysis has been utilized to evaluate the elemental composition of PLA nanocomposites. The intermolecular interaction and degradation temperature (*T*_d_) of 3D-printed blended polymer materials have been assessed using Fourier transform infrared spectroscopy (FTIR) and thermogravimetric analysis (TGA). The mechanical properties of the neat PLA and PLA/HAp blend polymer composite samples were analyzed through experimental testing, including tensile, compression, and flexural testing. It was concluded that the 12 wt % HAp blend with PLA polymer composite samples demonstrated great mechanical performance and interfacial strength characteristics. The tensile, flexural, and compression properties of PLA/12 wt % HAp were increased by 15.1, 10.05, and 13.19%, respectively, compared with neat PLA. The blended filament and fractography samples were analyzed by field emission scanning electron spectroscopy (FESEM).

## Graphical abstract

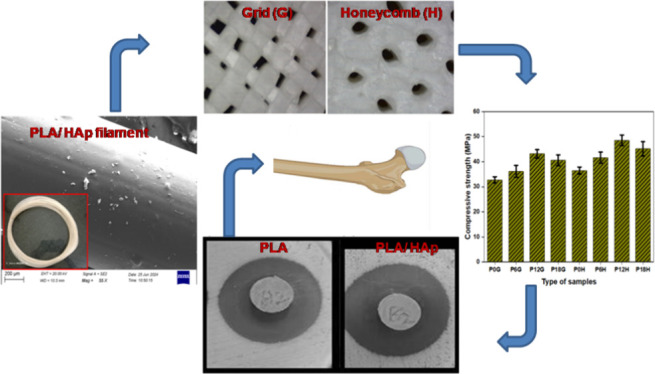

## 1 Introduction

Bone defects can develop from a variety of factors, including infections, congenital deformities, tumor excision, reconstructive surgery, and trauma, which can have severe detrimental implications on both the patient’s and society’s quality of life.^1^ Koushik et al. noted that most bone defects can resolve spontaneously when the physiological and environmental conditions are appropriate.^2^ However, many factors, including the size of the defect, biomechanical instability properties, wound’s environment, surgeon’s skill, patient’s hormone levels, patient’s diet, and patient’s level of stress, might prevent large defects from healing on their own.^3^ Therefore, bone tissue engineering has emerged as a potential new approach to overcome the current obstacles in bone repair and restoration.^4^ Also, substantial innovations in this sector are required to build functioning structures that allow for the regeneration of damaged bones.^5^ The predominant objective of regeneration is to develop new bone tissue that is similar to the original bone and might possess various features, such as immunological, structural, functional, and mechanical properties.^6^ Brunello et al. have demonstrated that new bone tissue might eventually grow to replace bone replacements; therefore, it is required to be biocompatible, bioresorbable, osteoconductive, and osteoinductive. Additionally, it needs to be safe, inexpensive, and simple to use.^7^ By supporting the tissue as it grows, a scaffolding structure offers one of the most promising ways to achieve the objectives of tissue engineering.^8^ Meanwhile, synthetic scaffolds face a challenge in satisfying the dual responsibility of bone tissue engineering scaffolds to facilitate bone regeneration by interacting with the local extracellular matrix (ECM) and providing load-bearing capability.^9^ Hence, suitable materials might be considered to meet these criteria, and researchers have noted that the utilization of biopolymers provides an alternative to conventional biocompatible materials (metallic and ceramic) and nonbiodegradable polymers.^10^

Among the most promising polymers is PLA, a thermoplastic that is biodegradable, renewable, and derived from fossil fuels.^11^ On the other hand, PLA’s biocompatibility, excellent processability, sufficient mechanical properties, and controlled degradation rate have driven substantial research into its potential use in bone tissue engineering (BTE) applications.^12^ Polylactic acid (PLA) has become a popular bone scaffold material in recent decades due to its inherent capacity to degrade through hydrolysis and enzymatic action upon implantation into the human body.^13^ However, the slow degradation of PLA can trigger a severe inflammatory reaction, potentially affecting cell proliferation and tissue repair. This could be due to the sudden release of acidic breakdown products or to their accumulation due to ineffective clearance from the scaffold’s surroundings.^14^ Some potential solutions that researchers have suggested to mitigate the acidic waste products and maintain a stable pH in the surrounding tissues involve incorporating biomaterials into the polymeric matrix and using low-molecular-weight PLA.^15^ Researchers have demonstrated that the incorporation of ceramic additives into the PLA matrix enhances mechanical characteristics, hydrophilicity, osteoconductivity, and mineralization upon implantation.^16^ In addition, bioceramic components act as buffers during the degradation process, thereby contributing to reducing acidic areas and balancing pH in the scaffold environment.^17^ Researchers are also considering bioceramics due to their exceptional osseointegration and osteoconduction properties, which aid in the regeneration of fractured and damaged bones.^18^

Furthermore, researchers have reported that hydroxyapatite (HAp) is a bioceramic material that is frequently utilized due to its exceptional bioactivity, biocompatibility, and osteoconductive properties. It has the potential to be used in clinical dentistry, tissue engineering, orthopedics, and maxillofacial surgery because the structure of HAp resembles an inorganic element of a bone matrix.^19^ It is utilized in bone/tissue regeneration due to its structural and chemical similarities to enamel, dentin, and bone.^20^ However, the poor mechanical properties of virgin HAp prevent its use in load-bearing implants.^21^ Also, researchers have reported that processing HA into the necessary form and shape is challenging due to its inherent hardness and fragility.^22^ On the other hand, pure HAp is exceedingly brittle, limiting its practical application. Consequently, HAp could be modified utilizing biodegradable polymers.^23^ Bone fillers made of nHA might be utilized for developing load-bearing bone repair scaffolds; it is easy to process, have strong cell activity, and could be utilized for neutralizing the acid produced by polymer degradation.^24^ It has also been noted that the surface of the pure HAp scaffold is rough, which restricts cell development and does not promote pseudopod, cell stretching, or proliferation.^25^ Akindoyo et al. have noted that HAp blended with PLA by injection molding methods provides better load-bearing properties and enhances biological and osteogenic performance, and it is crucially applied at the gradual replacement of bone tissue.^26^ The PLA phase may offer physical support for cell growth within these composites, while the HA phase may facilitate osteoinduction and cell proliferation.^27^ Senatov et al. found that adding 15% HAp increased the yield strength of the PLA/HAp scaffold significantly. Furthermore, no structural cracking or layer delamination could be seen during the initial compression test.^28^ The composite’s mechanical properties can be significantly enhanced by the addition of HA particles; however, the HA content in the composite was typically less than 20%.^29^ Furthermore, it was noted that the PLA/HAp scaffold exhibits a higher rate of shape recovery following compression cycles than the neat PLA samples.^30^ Russias et al. have demonstrated that the modulus requirements (usually 10–20 MPa for human cortical bone) might be achieved with HA concentrations of 10–40 wt %.^31^ Researchers discovered that 3D-printed PLA/HA did not induce the expression of inflammatory cytokines in dendritic cells, demonstrating no immunostimulatory properties, according to in vitro analysis.^32^ The porosity of the PLA/HA composite increases up to 15 wt % HA and then marginally decreases as the HA percentage increases. It was also found that the PLA/HA composite’s melting point initially decreased by up to 30% before gradually increasing. Conversely, the Tg of PLA/HA composites increases to 15% of HA before decreasing.^33^ The authors found that adding an excessive amount of HAp caused the components to be more porous and rough, which increased the surface area for the adhesion of cells. However, it also reduced the structure’s mechanical properties to a range that is comparable to cancellous bone.^34^ Additionally, the authors demonstrated that the scaffolds became more hydrophilic following the addition of HA through wettability analysis.^35^

Polymer nanocomposite composites can be processed by using a variety of methods, including the use of fillers. These methods include molding, solvent casting, in situ polymerization, and additive manufacturing.^36^ Three-dimensional printing is the most often utilized method for fabricating scaffolds because it allows for the development of objects with complicated geometry and regulated interconnected porosity.^37^ In the context of the current constraints associated with bone restoration, 3D-printed composite scaffolds have developed as a potential substitute.^38^ The printing of 3D scaffolds that are customized to the morphology and size of bone injury in a patient-specific and high-precision manner is facilitated by fused deposition modeling (FDM).^39^ Additionally, it was observed that additive manufacturing offers the potential to modify the design of the structures. This adaptability during the design phase enables the customization of the components to maximize their performance in the tissue engineering sector.^40^ It is feasible to develop 3D polymer composites that possess the necessary mechanical strength, biological properties, degradation rate, porosity, and pore size for bone tissue engineering.^41^ Furthermore, Maqsood et al. revealed that the infill pattern significantly influences the mechanical characteristics of the 3D-printed composites. Furthermore, it was discovered that the carbon fiber-reinforced PLA composite promoted improved mechanical performance for grid patterns compared to triangular infill patterns.^42^

Previous research has explored that bone tissue regeneration is an emerging area for bone damage and repair applications. It was reported that the PLA biodegradable polymer is predominantly used in medical applications. Also, researchers have found that hydroxyapatite (HAp) is frequently utilized as a bone filler for the fabrication of load-bearing PLA scaffolds for bone repair and restoration in the tissue engineering field. However, fewer studies are available to demonstrate the functional characteristics of PLA nanocomposites with a lower concentration of HAp (<20%), which could be used for cancellous bone applications. Also, HAp concentrations in PLA material play a significant role in promoting proliferation, cell adhesion, and differentiation, leading to better bone regeneration. Therefore, an investigation is required to evaluate the functional strength performance, physical properties, and biocompatibility behavior of PLA nanocomposites for biomedical bone regeneration applications. Moreover, infill patterns play a significant role in influencing the mechanical properties of bone scaffolds with desirable porosity levels. Hence, comprehensive investigation is required for various infill patterns for PLA/HAp composite bone structures fabricated by the material extrusion process.

The aim of this study is to investigate the mechanical strength and biocompatibility of PLA/HAp nanocomposite structures. The HAp nanoparticles have been blended with PLA at various concentrations, such as 6, 12, and 18 wt %, for cancellous bone regeneration applications. Further, 3D-printed various infill patterns (honeycomb and grid) are considered for promoting desirable mechanical properties, cell integration, and growth and enhancing bone regeneration in BTE application. Furthermore, the functional characterizations, such as mechanical (tensile, flexural, and compression), chemical (FTIR), and thermal (TGA) properties, are investigated on the PLA nanocomposites. Also, the physical properties (density and porosity) of PLA nanocomposites have been evaluated to facilitate bone ingrowth and vascularization. Moreover, antibacterial analysis has been utilized to explore the antibacterial properties of PLA nanocomposites. Field emission scanning electron microscopy (FESEM) investigates the surface morphology of blended composites and the fractography analysis.

## 2 Materials and Methods

### 2.1 Materials

The poly­(lactic acid) (REVODE290), which is easily accessible in the market, has a density of 1.24 g/cm^3^ and a melt flow index (MFI) of about 30 g/10 min (measured at 190 °C with a load of 2.16 kg). Nanohydroxyapatite particles (HAp) with a diameter of 50 and 80 nm were used in this study.

### 2.2 Filament Fabrication

Centrifugation was used to purify *n*-hydroxyapatite particles. After centrifugation, the supernatant and nHA were separated to form pellets at the bottom of the tube. Furthermore, acetone was mixed with nHA particles and subjected to an ultrasonic bath for 2 h to prevent particle agglomeration. Finally, nHA particles were mixed with PLA-dichloromethane solution, which was maintained on a magnetic stirrer for 2 h to produce well-dispersed PLA/HAp. After a week of evaporation at room temperature, the mixture was vacuum-dried to form the composite pellet PLA/nHA with variable concentrations of HA (6, 12, and 18 wt %). The single screw extruder, which develops 4 kg of filament per hour, was implemented for producing composite filaments. It allows for temperature regulation in accordance with the materials used in the filament fabrication process. A water cooling system on the extruder machine ensured the stability and output of the filaments. Additionally, we preheated the PLA/HAp composite pellets to 160 °C to achieve superior filament fabrication results. After drying, the composite granules were fed into a hopper, where they were melted and mixed to form a filament through extrusion. Sixty revolutions per minute and 210 °C were the extruder speed and nozzle temperature, respectively, employed in the extruder machine to produce composite filaments. A rapid cooling of the nozzle’s filaments is essential for improving their windability and rigidity. A gripper was employed to maintain the filament’s 1.75 ± 0.02 mm diameter during extrusion by applying tension. Figure 1 depicts the extruded PLA/HAp filament and the surface morphology of the PLA/18 wt % filament. It was shown that HA particles blend with PLA materials.

**Figure 1:**
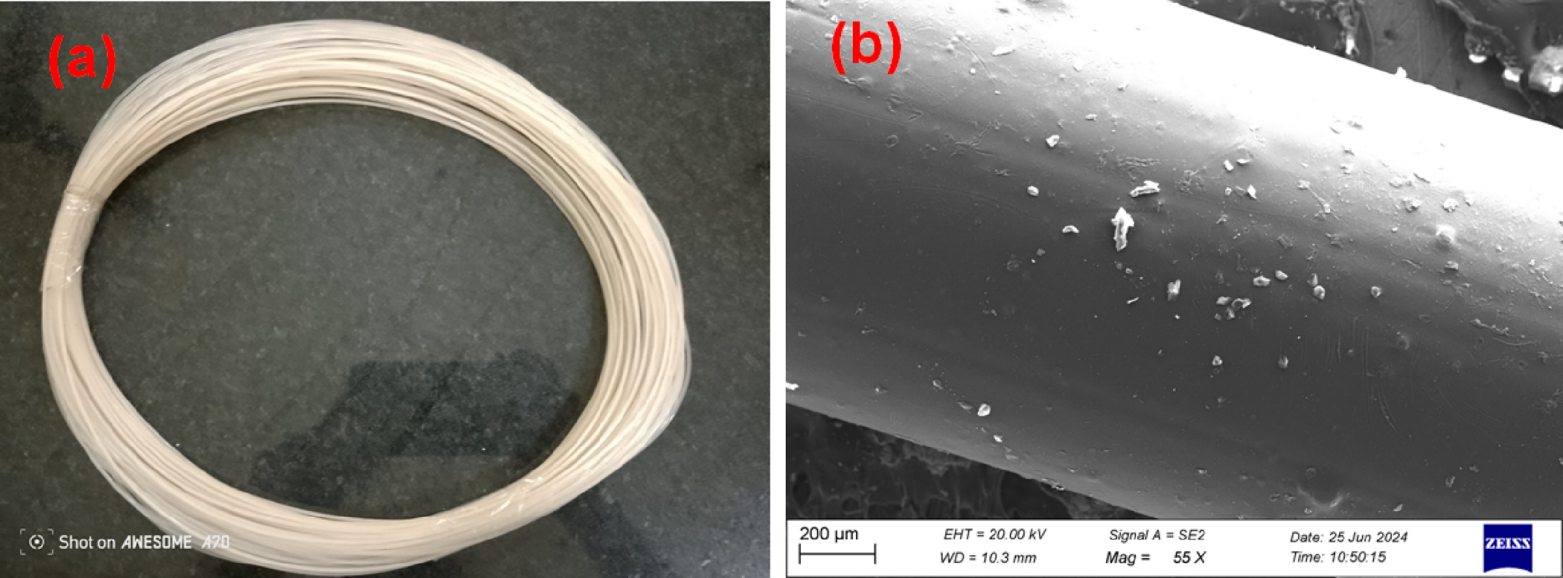
(a) Extruded PLA nanocomposite filaments. (b) SEM image of PLA with the 18 wt % HAp composite filament.

### 2.3 Sample Preparation

The extruded composite filaments were utilized for producing PLA and PLA/nHA samples using the FDM printer PRATHAM 3.0. The nozzle and bed temperatures were kept at 220 and 60 °C, respectively. The raster angle was kept at 0° to produce composite samples of high durability. The samples were developed by using CATIA V5R20 and exported as an STL slicing program to generate G codes. The G code functions as a compilation of directives supplied to the FDM machine, directing the execution of the procedure. Further, the composite samples were prepared for ASTM D695-6641, flexural samples were prepared for ASTM D790, and tensile samples were prepared for ASTM D3039, all in accordance with ASTM specifications. The following dimensions are specified for tensile, flexural, and compressive specimens according to ASTM standards: 50 × 8 × 2, 75 × 19, and 15 × 10 mm, respectively.^11^ Three samples of each type of material, such as neat PLA, 6% HA, 12% HA, and 18% HA, were printed and tested. These samples were produced as grid and honeycomb infill patterns to find a suitable pattern for BTE applications. The 3D-printed samples are shown in Table 1. The infill density was set to 70% for all of the types of configurations. The tensile, flexural, compressive, and density samples are shown in Figure 2.

**Table 1:** Types of Filaments and Specimen Codes

types of filaments	infill pattern	specimen code
PLA	honeycomb	P0H
PLA + 6 wt % HAp		P6H
PLA + 12 wt % HAp		P12H
PLA + 18 wt % HAp		P18H
PLA	grid	P0G
PLA + 6 wt % HAp		P6G
PLA + 12 wt % HAp		P12G
PLA + 18 wt % HAp		P18G

**Figure 2:**
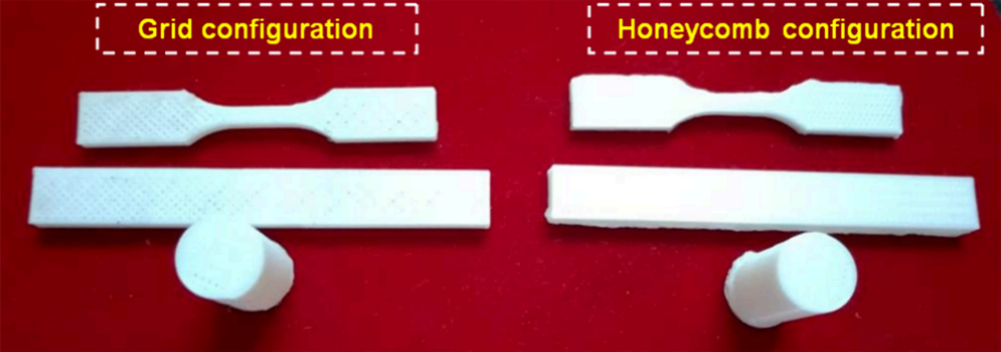
Sample preparations for experimental testing analysis.

### 2.4 Experimental Characterization

The mechanical tests were carried out using a Universal Testing Machine (UTM-Tinius Olsen), which has a maximum load cell capacity of 50 kN and a strain rate of 1 mm/min. The samples were subjected to tensile, compression, and flexural testing. Additionally, the molecular structure and functional groups of polymer materials were determined using FTIR.^45^ The chemical bonds and structure of pure polymer and blended polymer samples were analyzed using the Spectrum Two FTIR/Sp10 Software (PerkinElmer, USA). Moreover, actual densities (ρ_a_) of PLA nanocomposite samples were determined by the Archimedes method with water as the immersion liquid. The density was calculated as in eq 1. Also, the theoretical density was determined by using eq 2.^33^

$$\rho_{a}=\frac{\mathrm{mass}\;\mathrm{in}\;\mathrm{air}}{\left(\mathrm{mass}\;\mathrm{in}\;\mathrm{air}-\mathrm{mass}\;\mathrm{in}\;\mathrm{water}\right)}\times \mathrm{density}\;\mathrm{of}\;\mathrm{water}$$ 1

$$\rho_{t}=\left(d_{P}\times W_{P}\right)+\left(d_{H}\times W_{H}\right)$$ 2

$$\mathrm{porosity}\left(\%\right)=1-\frac{\mathrm{actual}\;\mathrm{density}}{\mathrm{theroetical}\;\mathrm{density}}\times 100$$ 3

where ρ_a_ is the actual density (g/cm^3^), ρ_t_ is the theoretical density (g/cm^3^), *W*_p_ is the weight of PLA (grams), *W*_H_ is the weight of HAp (grams), *d*_p_ is the density of PLA (g/cm^3^), and *d*_H_ is the density of HAp (g/cm^3^). Also, the porosity (P) was calculated using eq 3. A thermogravimetric analyzer was used to measure TGA analysis for PLA and PLA nanocomposites. The alumina crucibles were filled with the samples (7.5 ± 0.5 mg), and a reference crucible that was empty was utilized. A blank measurement was taken and then subtracted from the experimental to remove the buoyancy effect.^43^ The PLA nanocomposites were heated at a rate of 15 °C/min in a flow of 50 mL/min of N_2_. The temperature range was room temperature to 600 °C. The temperature, mass, and first derivative of the sample as well as the heat flow were recorded continuously. Furthermore, the antibacterial test was performed on PLA and PLA nanocomposites for determining the antibacterial activity using the disc diffusion method and the colony-forming unit (CFU). In this study, *Staphylococcus aureus* (*S. aureus*) was utilized for wound infection and regeneration processes. Before the experimental work, the PLA and PLA/HAp scaffolds were cut into a disc shape and sterilized with ultraviolet radiation for 40 min. The bacteria were uniformly cultured on Petri plates in an agar medium by using a spread plate technique for qualitative analysis. The sterilized discs were incubated in agar at 40 °C for 24 h, and the bactericidal effect of the samples was determined by capturing the area of inhibition zones with a digital camera. For quantitative analysis, the standard spread plate method was employed to inoculate approximately 0.1 mL of the test extract with bacteria over sterilized nutrient agar medium plates. The plates were incubated in a CO_2_ incubator for 24 h. Additionally, a digital colony counter was employed to determine the average number of CFUs.

Furthermore, the cell viability of the 3D-printed PLA and PLA/HAp nanocomposite scaffolds was assessed using MG63 human osteosarcoma cell lines^61^ in the MTT assay. The cells were cultured in Dulbecco’s modified Eagle’s medium (DMEM) that contained 10% fetal bovine serum (FBS) and 1% penicillin–streptomycin supplement.^46^ The cells were maintained at 37 °C under humidified conditions with 5% CO_2_. The scaffold samples were sterilized by immersing them in 70% ethanol for 30 min on each side followed by a 30 min exposure to UV light and a subsequent rinse in sterile PBS. The scaffolds were inoculated with MG63 cells at a density of 5 × 10^5^ cells per well in a 96-well culture plate after the sterilized samples were transferred. Cell attachment and growth were determined in the plate after 24 and 48 h of incubation. Following the incubation period, 50 μL of the 3-(4,5-dimethyl-2-thiazolyl)-2,5-diphenyl-2*H*-tetrazolium bromide (MTT) reagent was added to each well and incubated at 37 °C for 4 h. The Vmax Microplate reader was used to measure the absorbance at 570 nm after 200 μL of dimethyl sulfoxide (DMSO) incubated mixture was added to a 96-well plate. The relative growth rate (RGR) relation (eq 4) was used to determine the cell viability as a percentage in comparison to the control sample groups.^62^

$$\mathrm{RGR}=\frac{{\mathrm{OD}}_{\mathrm{Sample}}}{{\mathrm{OD}}_{\mathrm{Control}}}\times 100$$ 4

where OD_Sample_ is the average optical density value of each sample and OD_Control_ is the average optical density of the control sample.

## 3 Results and Discussion

### 3.1 Surface Morphology and Elemental Analyses

Figure 3 depicts the SEM and EDAX investigations of PLA nanocomposites. It has been found that n-HAp is distributed equally throughout the surface morphology of PLA nanocomposites. However, as the content of HP particles increased, substantial agglomerations were detected. This could be because increased HA content promotes higher surface energy between PLA and HP particles, lowering PLA–nHA interfacial contact and causing nHA particle aggregation in the polymer matrix.^44^ Moreover, a homogeneous dispersion of HAp particles was confirmed from a morphological perspective alongside an increase in surface roughness. EDAX analysis further revealed that higher calcium and phosphorus levels indicated the addition of additional HAp^45^ to the PLA matrix.

**Figure 3:**
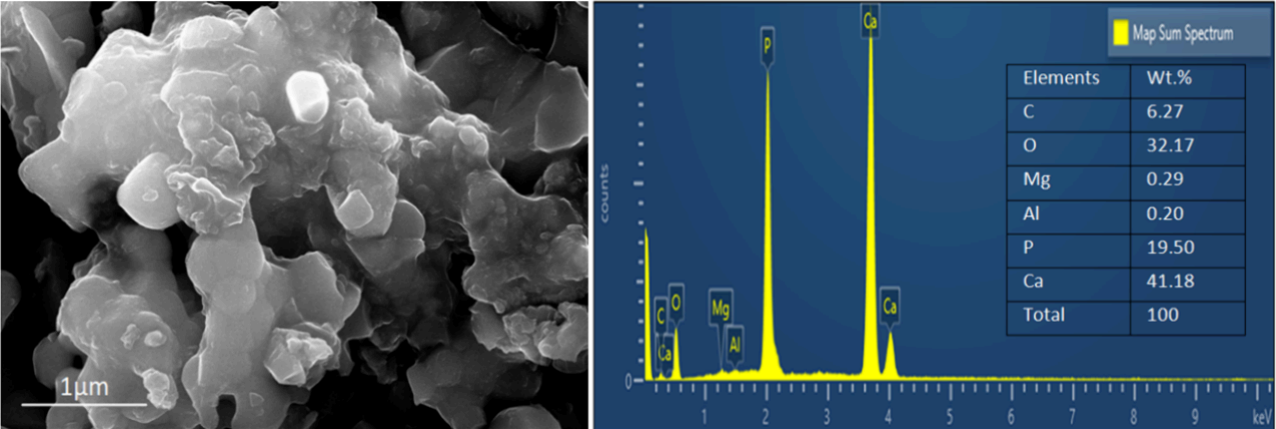
SEM (magnification at 10,000×) and EDAX analysis of PLA/18 wt % HAp.

### 3.2 FTIR Analysis

The FTIR spectra of PLA, HAp, and PLA/HAp nanocomposites are shown in Figure 4. The strong peak at 1754 cm^–1^ shows the C=O stretching vibration of the carbonyl and ester groups for PLA materials. Furthermore, it was discovered that the C=O peak was slightly shifted at 1749, 1746, and 1739 cm^–1^, corresponding to 6, 12, and 18 wt %, respectively. It was evident that HAp had been incorporated with PLA in an appropriate quantity. The C–O–C stretching ester group is represented by the peak at 1180 cm^–1^. C–O stretching vibrations and C–H groups are also introduced, with peaks at 1078 and 858 cm^–1^, respectively. Additionally, C–H bending bonds are exhibited at 716 cm^–1^. Furthermore, PLA contains polar functional hydroxyl groups (−OH), which are crucial to its reactivity and compatibility with other materials. The stretching band at 3040–2800 cm^–1^ represents both asymmetric and symmetric stretching of ethyl and ethylene’s C–H. It is to be noted that the PLA spectra show a very high intensity of this band, which is due to the comparatively high concentration of ethylene components in PLA.^46^ Further, peaks coming from HAp are generally from phosphate groups, such as 1090, 1025, 606, and 551 cm^–1^ (PO_4_^3–^ bending) and 960 cm^–1^ (PO_4_^3–^ stretching). Also, it was noted that the PO_4_ bending peaks (O–P–O) were introduced significantly at 552 cm^–1^ in all PLA/HAp FTIR spectra. Additionally, it was found that the P–O stretching vibration of the phosphate group ranged from 1030 to 1088 cm^–1^ in all PLA nanocomposite samples. It has been demonstrated that HAp is successfully synthesized with the PLA material and that hydroxyapatite plays a key role in increasing the biocompatibility, functional interactions, and mechanical properties of PLA/HAp nanocomposites.^47^

**Figure 4:**
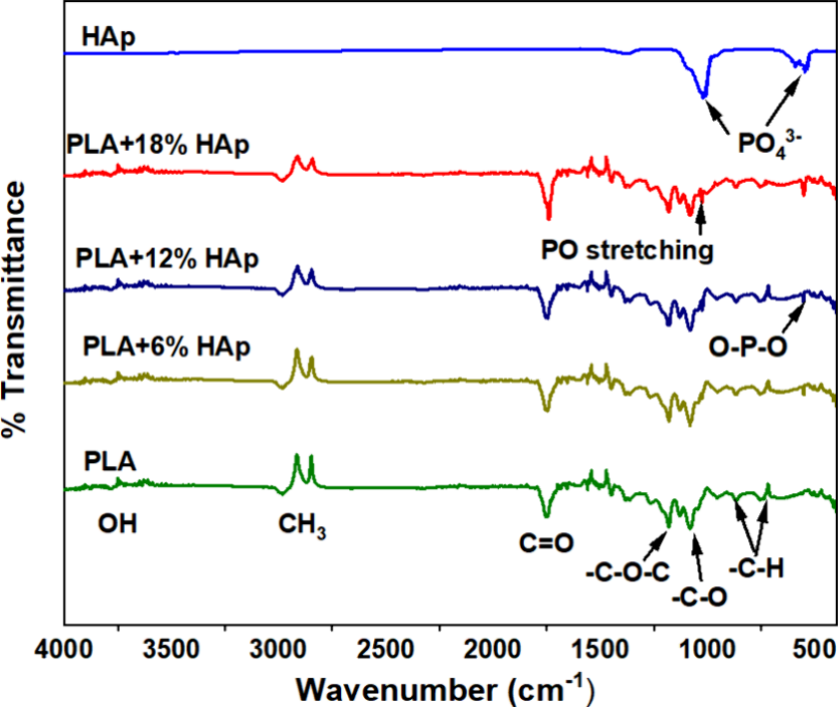
FTIR analysis of PLA and PLA nanocomposites.

### 3.3 Thermal Analysis

Thermogravimetric (TGA) analysis was performed on the filaments to assess the thermal stability of the composites and the percentage of HA in the filaments following the polymer extrusion process.^35^ Figure 5 illustrates the TGA analysis of PLA and PLA/HAp nanocomposite filaments. It was observed that the thermal decomposition temperature was between 20 and 700 °C. It was found that the degradation temperature is slightly increased with an increase of HAp concentration with PLA materials. Also, it was noted that the degradation temperature of PLA materials was observed as 375 °C. Further, the degradation temperature was significantly increased while PLA blended with HAp materials. This might be due to HAp raising the initial temperature of decomposition and the polymer activation energy in PLA composites.^48^ Also, it was indicated that the thermal stability has been significantly enhanced with the increase of HAp in PLA nanocomposites. It has been proven that the thermal stability of a composite is influenced by the strength of interfacial bonding. It is reported that the interfacial interaction in HA-based polymer composites is significantly enhanced by the electrostatic binding between the carboxylate group of polymers and the Ca2^+^ ions of HA.^26^ Also, Tazibt et al. found that better dispersion of HAp particles in PLA composites improves interfacial bonding between polymer and particles, which is attributed to increasing the degradation temperature in PLA polymer composites.^49^ Moreover, it was observed that the highest loss was observed in the PLA material, whereas no solid residue is obtained in the PLA material. Also, the residue materials are obtained in all the types of PLA nanocomposites at less than 700 °C. It was observed that the HAp residue composition varies in composite filaments because HAp particles might accumulate within the polymer matrix.^35^ The degradation temperature and residue materials are illustrated in Table 2.

**Figure 5:**
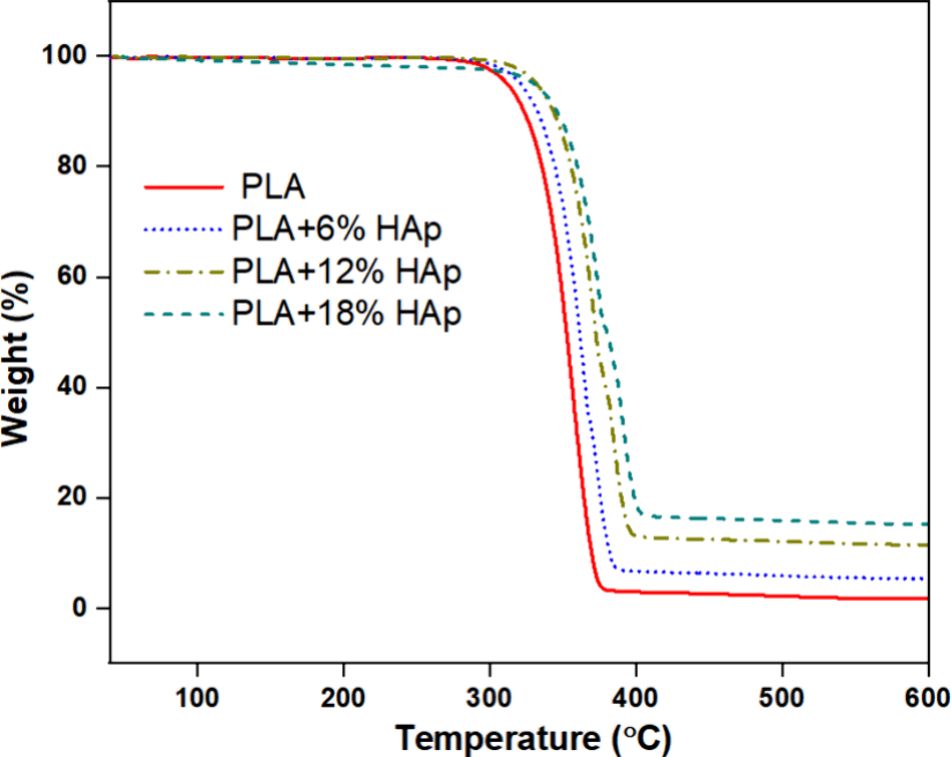
TGA analysis of PLA and PLA nanocomposite filaments.

**Table 2:** Percentage of Residue Materials and Degradation Temperature of 3D-Printed Samples

sample	residue %	degradation temperature (°C)
PLA	0	375
PLA + 6% HAp	4.63	384
PLA + 12% HAp	10.72	395
PLA + 18% HAp	16.48	401

### 3.4 Density and Porosity Analysis

The density and porosity of PLA and PLA nanocomposite samples have been evaluated for use in bone tissue engineering applications. The biomaterials were extruded to form a continuous filament that contained PLA with 6, 12, and 18 wt % of HAp. This filament was used for producing honeycomb- and grid-shaped porous scaffolds with FDM. Figure 6 shows that increasing HAp concentrations greatly increases the actual density of porous scaffolds. This could be owing to the homogeneous distribution of nanoparticles during the extrusion of composite filaments. Furthermore, it was discovered that the honeycomb pattern scaffold structure had a higher density than the grid infill pattern at the same HAP concentration. It shows that the density of PLA nanocomposites varies from 1.23 to 1.28 g/cm^3^, which is most comparable to the density of human cortical bone.^50^ Figure 6 also shows an investigation into the porosity of PLA and PLA nanocomposites. The honeycomb pattern has a lower porosity than the grid structure. This could be because honeycomb geometries have a denser configuration compared with grid structures. Furthermore, the honeycomb pattern occupied more solid areas than the grid scaffold design, resulting in a superior mechanical performance compared to the grid scaffold.^51^ Table 3 illustrates the actual, theoretical density and porosity of the honeycomb and grid scaffold 3D-printed structure. It was found that the structure’s ultimate porosity was only 18.59% for grid composite structures. Despite this, the porosity falls below the recommended range (30%) in the literature for cancellous bone regeneration.^52^ Also, the implant’s porous structure produces channels that facilitate bone growth. Further, porosity contributes to the biological interaction between bone and implant.^53^ Moreover, the pore size was evaluated by using an optical microscope. The optical images are captured at 25× magnification to show the pore size in the printed PLA composite samples. It was found that the pore size of 3D-printed PLA/HAp 18 wt % scaffolds is 800 μm, which is depicted in Figure 7.

**Figure 6:**
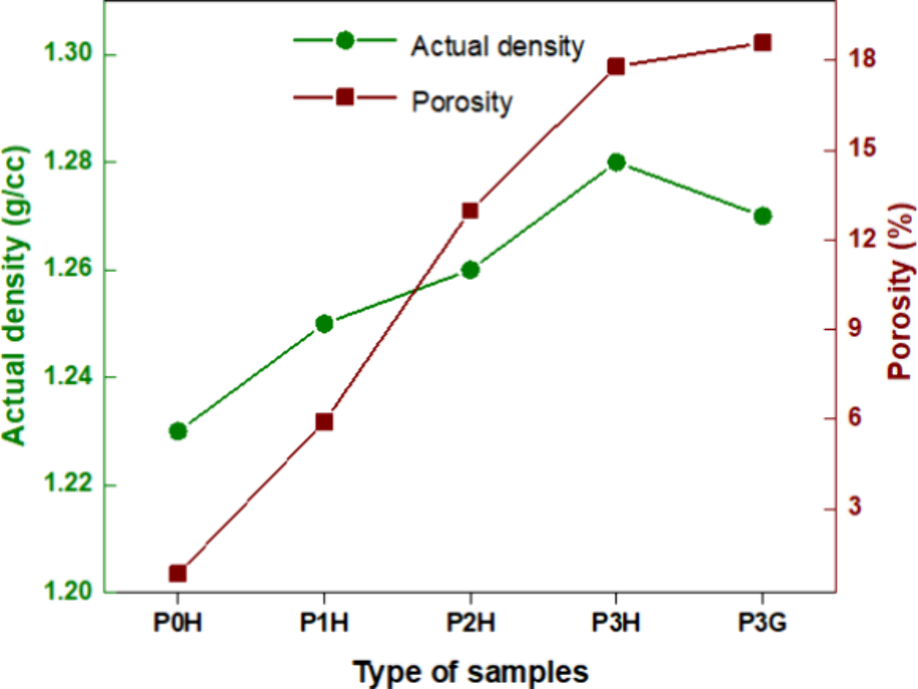
Density and porosity of 3D-printed samples of honeycomb and grid geometries.

**Table 3:** Density and Porosity of PLA Nanocomposites

sample	actual density (ρ_a_) (g/cm^3^)	theoretical density (ρ_t_) (g/cm^3^)	porosity (%)
P0H	1.23	1.24	0.84
P1H	1.25	1.33	5.92
P2H	1.26	1.45	12.98
P3H	1.28	1.56	17.80
P3G	1.27	1.56	18.59

**Figure 7:**
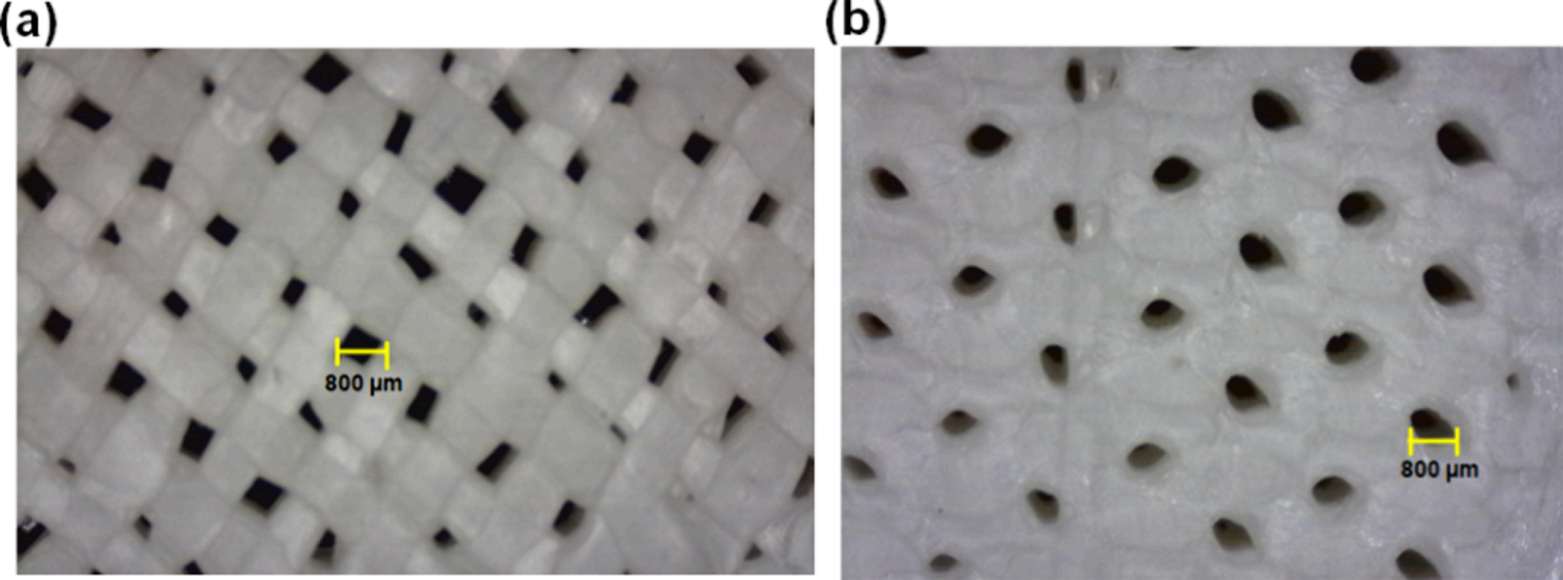
Pore size of the (a) grid and (b) honeycomb PLA composite structure.

### 3.5 Mechanical Strength Characterization

#### 3.5.1 Tensile Properties

Figure 8a–c illustrates the tensile characteristics of PLA and PLA nanocomposites in various configurations. Figure 8a depicts the tensile strength of the honeycomb infill pattern analysis. It was found that HAp concentrations have a significant effect on tensile strength in the PLA material. It was noted that the tensile strengths for P0H, P6H, P12H, and P18H composites are 36.92 ± 0.69, 43.23 ± 0.42, 51.38 ± 0.48, and 49.1 ± 0.54, respectively. The tensile strength of P12H was found to be 28.14% higher than that of pure PLA materials. Moreover, P18H exhibits a 4.4% reduction in tensile strength when compared with P12H composite samples. This could be attributed to reduced interfacial adhesion between the matrix and nanomaterials. On the other hand, increasing the HAP concentration causes aggregation in PLA composites, resulting in a decrease in the tensile strength of the PLA/HA composites.^54^ Figure 8b shows the tensile strength of the grid pattern configuration. The grid infill pattern configuration follows a similar trend in which the tensile strength increases with increasing HAp concentrations in PLA material. The tensile strengths of P0G, P6G, P12G, and P18G composites are 32.8 ± 0.7, 36.7 ± 0.58, 45.49 ± 0.52, and 43.7 ± 0.86, respectively. It was reported that the tensile strength of P12H increased by 27.9% compared with that of the pure PLA material. This might be due to the inclusion of HA particles in PLA composites preventing the material from entering the zone of permanent deformation.^50^ Additionally, it was noted that the tensile curve of honeycomb and grid PLA nanocomposites is gradually decreasing, which is indicative of a more effective energy absorption and stress redistribution before the material’s complete failure. It has been established that printing of these infill structure composites is free of defects.

**Figure 8:**
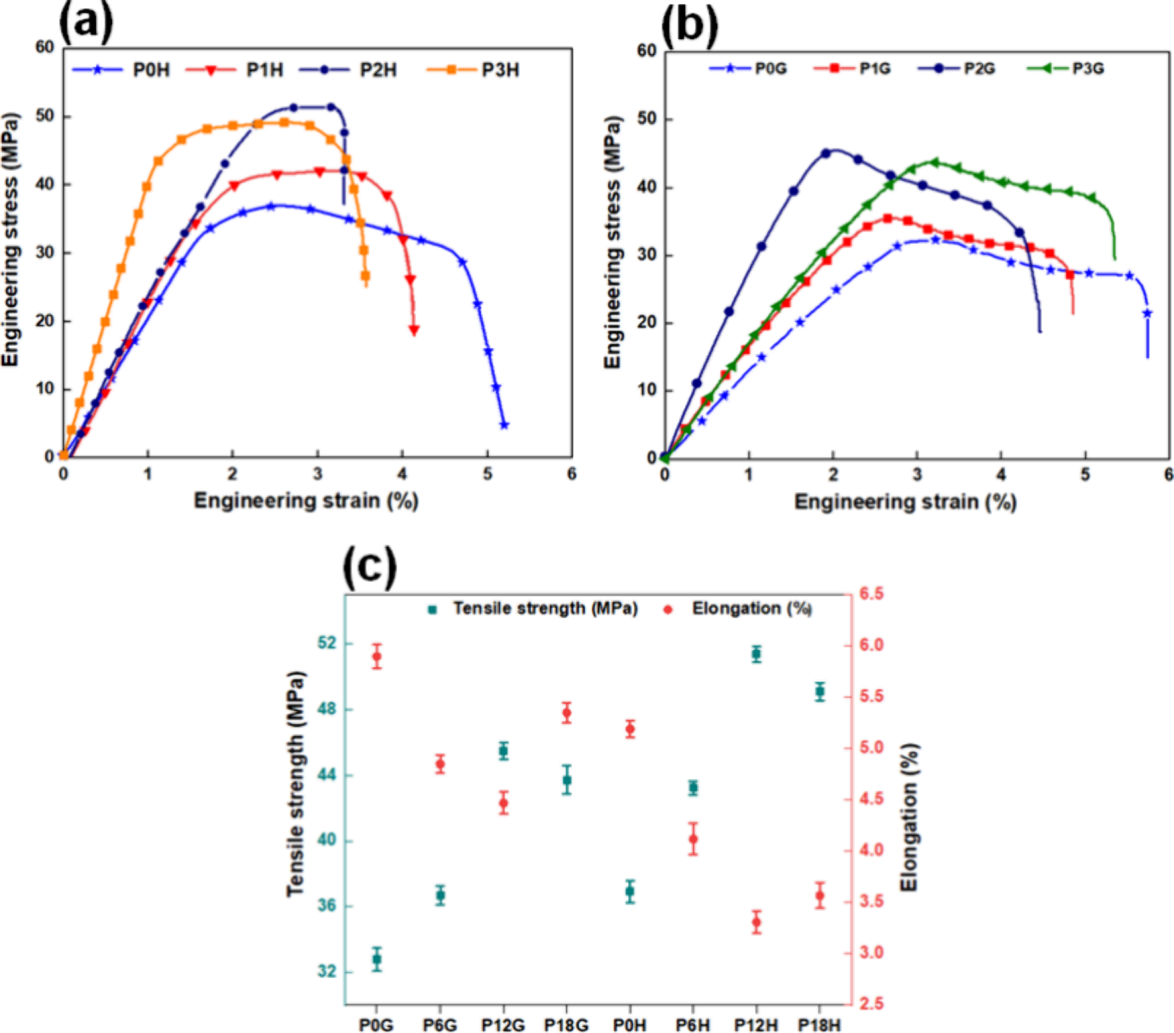
Tensile strength analysis of the (a) honeycomb configuration and (b) grid configuration and (c) tensile strength and elongation analysis.

Figure 8c depicts the tensile strength and elongation for the honeycomb and grid PLA nanocomposites. It was discovered that honeycomb structures have significantly higher tensile strength than grid pattern composites. However, honeycomb pattern composites have substantially lower elongation than grid design composites. This could be because denser honeycomb composite structures provide more load-bearing capacity in the composites. It may also improve uniform stress distribution, resulting in fewer premature failures of honeycomb composite structures. Moreover, denser honeycomb composites improve energy transmission between cells, delaying crack initiation and propagation and thereby enhancing the honeycomb structure’s tensile strength.^55^ Furthermore, the elongation of the P0G grid pattern is increased by 12% compared to the POH honeycomb sample. This might be due to the load being transmitted through the linear struts of grid cell structures, which could elongate in the direction of the tensile load. In contrast, the load is transmitted in multiple directions, which can lead to a more rigid response and less elongation under similar conditions^51,56^

#### 3.5.2 Bending Properties

Figure 9 shows the flexural strength properties of various configurations of PLA and PLA composites. The flexural strengths of grid configurations are 36.7 ± 1.3, 49.2 ± 2, 62.8 ± 1.4, and 59.1 ± 2.3 for P0G, P6G, P12G, and P18G respectively. Similarly, the flexural strengths of honeycomb configurations are 42.4 ± 2.1, 54.7 ± 2.6, 67.3 ± 1.8, and 65.7 ± 2.7 for P0H, P6H, P12H, and P18H, respectively. It was noted that flexural strength is prominently enhanced by increasing the HA concentration in PLA materials. This is due to the fact that a larger concentration of HA inhibits the polymer’s ability to flow effectively, which is essential for the formation of a strong bond in the polymer mixture.^50^ However, the addition of 18 wt % HA particles to the PLA material reduces flexural strength in both configurations. Also, it was found that the flexural strength of the P12H honeycomb sample increased by 6.7% compared with the P12G grid sample. The PLA nanocomposite’s flexural strength was enhanced in the honeycomb structure due to the prevention of crack initiation at the interface of cells.^50^ Hence, the strength produced by the 3D-printed PLA composite structure might be higher than the cancellous bone^57^

**Figure 9:**
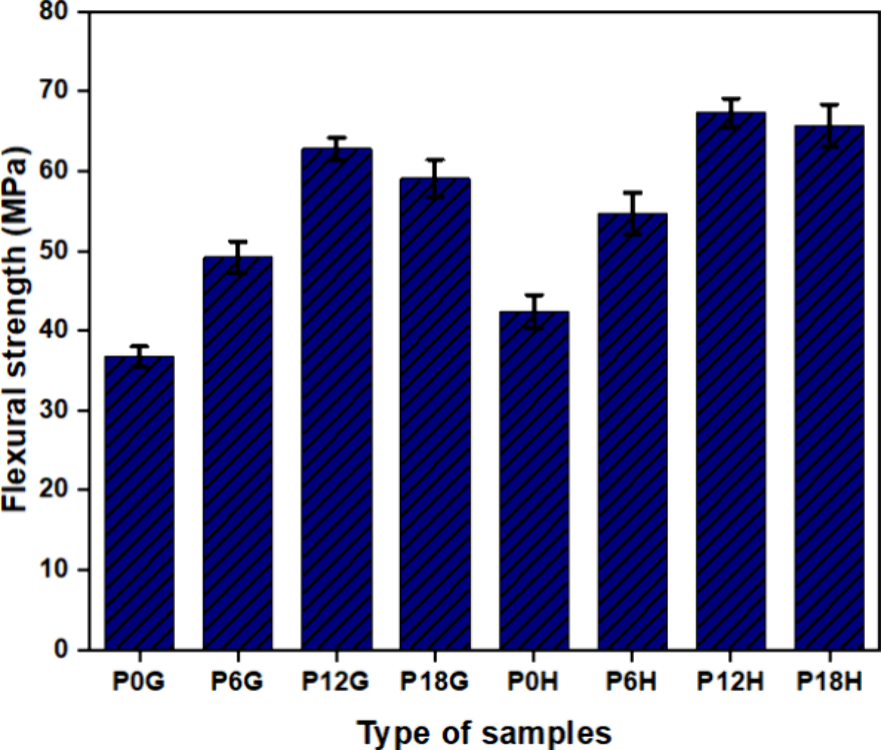
Flexural strength analysis of PLA and PLA nanocomposites.

#### 3.5.3 Compressive Properties

The compressive characteristics of 3D-printed PLA and PLA nanocomposites for various infill patterns are investigated. Figure 10 shows the compressive strengths of honeycomb and grid composite structures. The compressive strengths of grid configurations are 32.8 ± 1.25, 36.2 ± 2.36, 43.2 ± 1.7, and 40.6 ± 2.16 for P0G, P6G, P12G, and P18G respectively. Similarly, the compressive strengths of honeycomb configurations are 36.5 ± 1.37, 41.7 ± 2.2, 48.6 ± 2.1, and. 45.2 ± 2.83 for P0H, P6H, P12H, and P18H, respectively. It was noted that the compressive strength of the honeycomb composite structure is significantly higher than the grid composites. This might be due to the grid composite structure having slightly higher porosity compared with honeycomb structures.^40,58^ Also, it was found that the increase in the concentration of HA particles leads to an enhancement of the compressive properties in PLA composites because higher nHA results in a greater number of convex and concave surfaces on the scaffold, increasing the scaffold’s surface area. An increase in surface area corresponds to an increase in the amount of space set aside for cell growth, which promotes cell growth and proliferation.^39,59^ In contrast, the compressive properties of the composite were found to decrease as the proportion of nHA increased, resulting in a rougher filament and scaffold surface. However, this was still significantly higher than that of pure HA porous ceramic (approximately 5.5 MPa) and natural human cancellous bone (1–12 MPa). Consequently, the rougher scaffold surface might limit cell proliferation, stretching, and pseudopod growth.^25^ Moreover, according to Koushik et al, the scaffolds for reconstructing bone defects demonstrated compression strengths between 1.5 and 45 MPa, which is the range of human trabecular bone, at higher porosities.^2^

**Figure 10:**
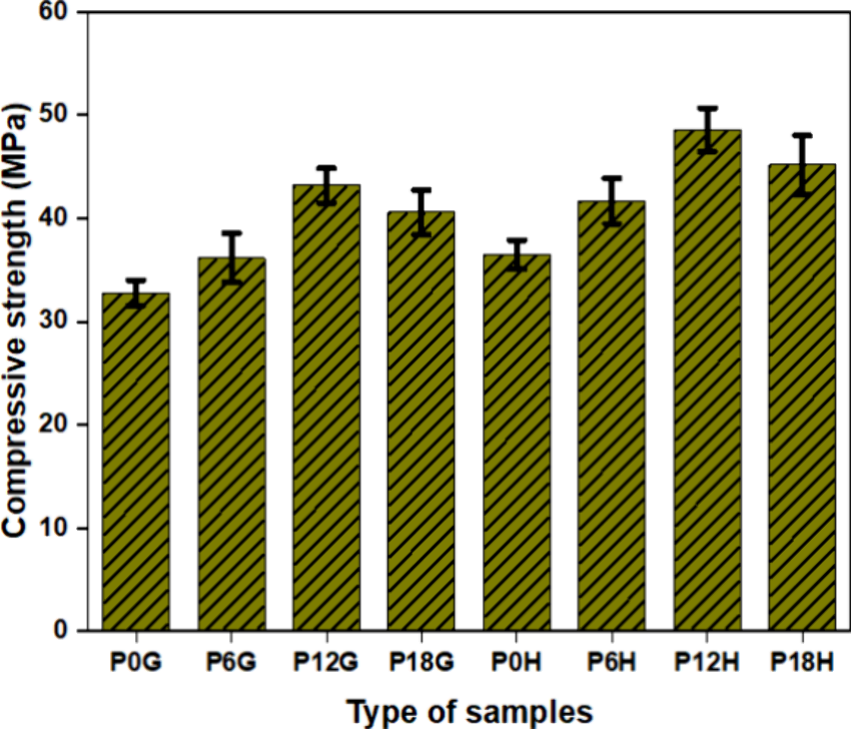
Compressive strength analysis of PLA and PLA nanocomposites.

### 3.6 Antibacterial Analysis

The qualitative and quantitative antibacterial activity of the PLA and PLA/HAp composites was evaluated against *S. aureus* using the zone of inhibition, as illustrated in Figure 11a,b, and the CFU method, as illustrated in Figure 11c,d. Figure 11a shows the lower inhibition zone (the dark region surrounding the sample), which indicates that pure PLA has a low antibacterial effect. Figure 11b demonstrates that the PLA composite sample had a higher inhibition zone, indicating that the PLA/Hap scaffold was more efficient against bacterial inhibition. The addition of the composite increases the antibacterial activity, as evidenced by the larger dark region. This could be attributed to the release of active oxygen groups from HAP, which could demonstrate antibacterial properties in the composites.^60^

**Figure 11:**
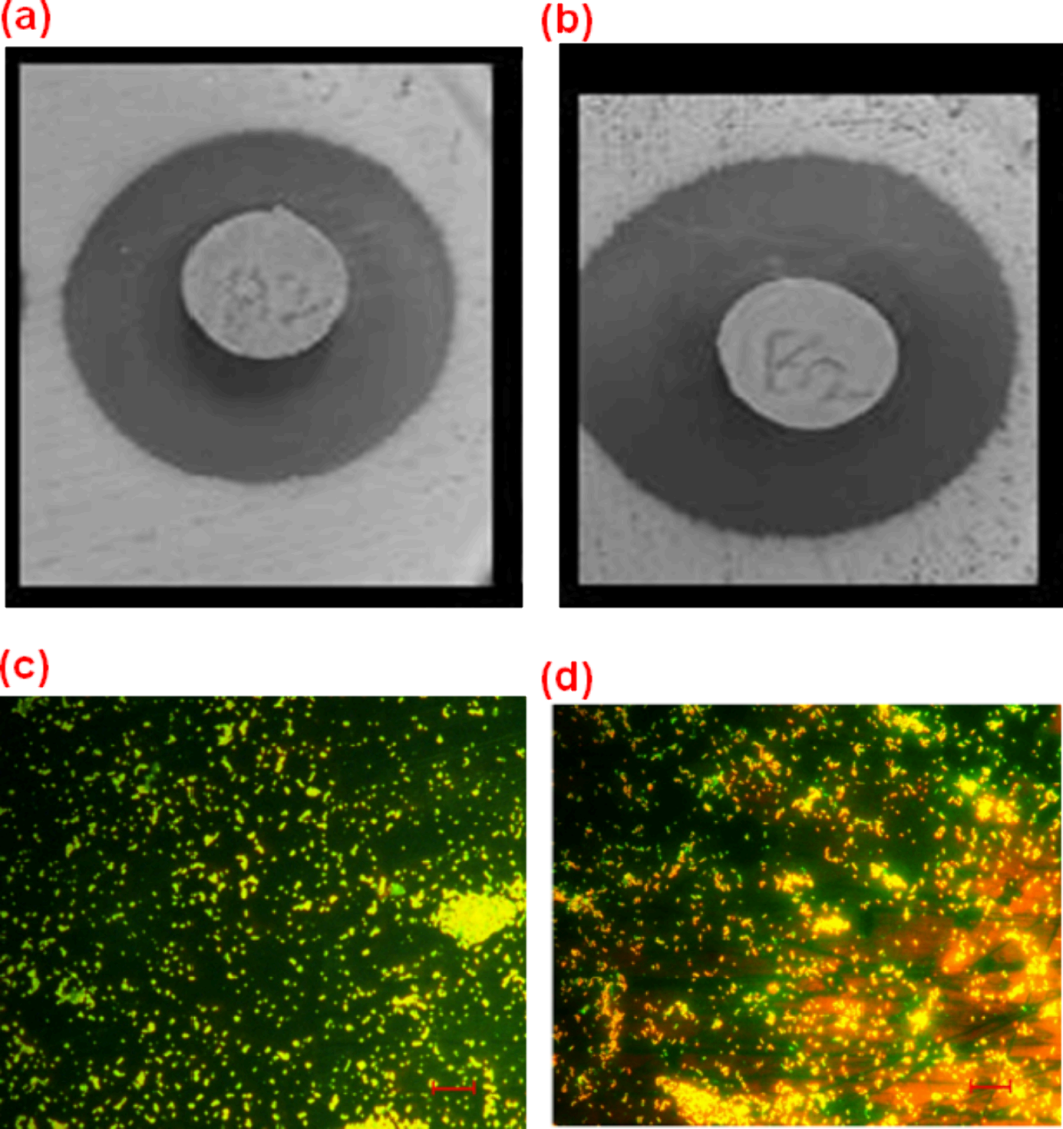
Qualitative antibacterial assessment of the (a) PLA^66^ and (b) PLA/HAp scaffold. Quantitative antibacterial assessment of the (c) PLA^66^ and (d) PLA/HAp scaffold.

Figure 11c indicates a denser distribution of bright areas, which implies a higher probability or extent of bacterial colonization on the PLA surface. It was noted that the pure PLA material is less effective in inhibiting the growth of bacterial colonization. However, fewer dispersed bright bacterial spots are indicated in Figure 11d. The reduced bacterial colonies indicate that PLA/HAp composites provide better antibacterial properties. The various colors may also indicate the areas in which bacterial growth has already been inhibited, indicating that the antibacterial PLA/HAp material composites are efficiently active in composites. The CFUs measured for PLA and PLA nanocomposites are 5.7 × 10^3^ and 1.9 × 10^3^ CFU/cm^2^, respectively.

### 3.7 MTT Assay

The cell viability of the 3D-printed PLA and PLA/HAp nanocomposite scaffolds was assessed by using the MTT assay. The cell viability has been observed within 2 days of culture at 24 and 48 h. Figure 12 shows that cell viability is greatly increased in the PLA/HAp nanocomposites compared to the PLA material due to the significant infiltration of HA particles in the PLA matrix. The cell viability of PLA and PLA/18 wt % HAp composites was 81.4 ± 2.43 and 88.3 ± 1.94%, respectively, after 48 h of culture. After 24 h of culture, PLA and PLA/18 wt % HAp composites had cell viability rates of 80.5 ± 2.18 and 86.5 ± 1.87%, respectively. It was also shown that increasing the duration spent in the cell culture improves cell attachment. The cell compatibility was proven by testing the PLA/HAp scaffold in a lab with MG63 cells, revealing that more than 70% of the cells were alive, which was attributed to the higher biocompatibility of composites,^63^ and it could be utilized for biomedical applications. The PLA/HAp nanocomposites exhibit increased cell viability at higher concentrations of HAP, which serves as evidence that this composition is a significant material for the rejuvenation and regeneration of bone tissues.^64^

**Figure 12:**
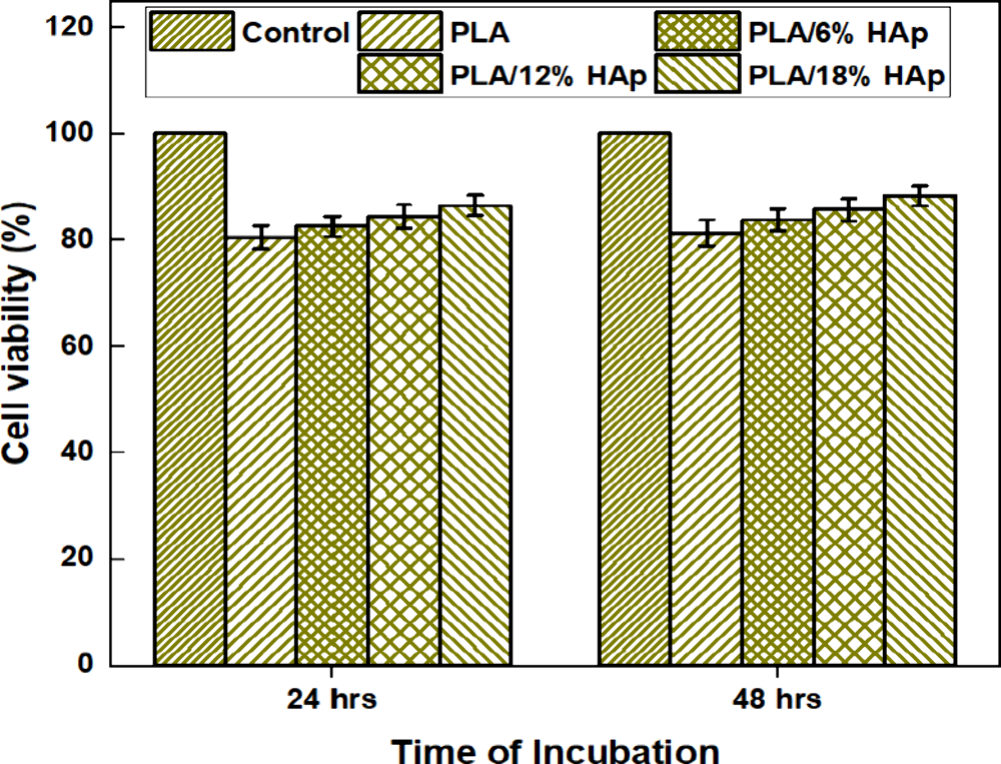
Percentage of cell viability of control and PLA nanocomposites.

### 3.8 Fractography Analysis

The dispersion of HAp in the PLA matrix is crucial for the resultant composite to exhibit a satisfactory mechanical performance. The bonding strength at the interfacial boundaries of the PLA and HAp is recognized to be extremely important for acceptable mechanical characteristics in PLA nanocomposites.^26^ Figure 13 shows fracture surface morphologies of PLA with 6 and 12 wt % nanocomposites. Figure 13a shows that the surface is relatively smooth, which was produced by the brittle PLA matrix with minimal plastic deformation. Figure 13b indicates that HA nanoparticles are incorporated uniformly into the PLA matrix. Also, it was observed that there was better interfacial bonding between the PLA matrix and HAp particles. This might be due to crack bridging and matrix shear-yielding mechanisms being involved to promote better interfacial boning in PLA nanocomposites.^26^ Also, the ceramic phase is bonded to the polymer matrix, which imparts the material’s strength and durability.^31^ Moreover, an exfoliated structure is observed by increasing the concentration of HA particles. It was noted that the filler and polymer matrix are well-organized, which promotes the enhancement of the mechanical strength of PLA nanocomposites.^46^ It was reported that by adding HA to the PLA blend, the material’s bioactivity and biocompatibility may be increased, increasing its ability to promote bone formation and improve tissue adhesion.^65^

**Figure 13:**
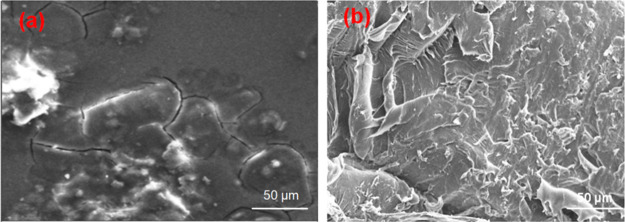
Surface morphology of PLA nanocomposites: (a) 6 wt % and (b) 12 wt % of HAp.

## 4 Conclusions

This research focused on 3D-printed PLA nanocomposite structures for bone tissue engineering (BTE) using varying concentrations (6, 12, and 18 wt %) of hydroxyapatite (HAp) nanoparticles blended with PLA via material extrusion. Grid and honeycomb structures were fabricated to evaluate mechanical, degradation, microstructural, and antibacterial properties. Honeycomb PLA composites exhibited superior tensile, flexural, and compressive strengths (by 11.16, 13.4, and 10.14%, respectively) due to lower porosity and higher density compared with the grid structure. FTIR and EDAX confirmed the successful HAp incorporation, while TGA showed a 6.93% increase in the degradation temperature, indicating enhanced thermal stability in the PLA nanocomposite scaffold. PLA nanocomposites also showed improved antibacterial activity and biocompatibility. Among the blends, PLA with 12 wt % HAp demonstrated optimal mechanical and thermal performance. However, excessive HAp (18 wt %) reduced mechanical properties due to agglomeration and weak particle–matrix interaction. The findings suggest that honeycomb-structured PLA nanocomposites have significant potential for applications in human trabecular and cancellous bone.
